# Telemonitoring and Mobile Phone-Based Health Coaching Among Finnish Diabetic and Heart Disease Patients: Randomized Controlled Trial

**DOI:** 10.2196/jmir.4059

**Published:** 2015-06-17

**Authors:** Tuula Karhula, Anna-Leena Vuorinen, Katja Rääpysjärvi, Mira Pakanen, Pentti Itkonen, Merja Tepponen, Ulla-Maija Junno, Tapio Jokinen, Mark van Gils, Jaakko Lähteenmäki, Kari Kohtamäki, Niilo Saranummi

**Affiliations:** ^1^Eksote, South Karelia Social and Health Care DistrictLappeenrantaFinland; ^2^VTT Technical Research Centre of FinlandTampereFinland; ^3^Mawell Care OyHelsinkiFinland; ^4^Medixine LtdHelsinkiFinland

**Keywords:** health coaching, telemonitoring, type 2 diabetes, heart disease, personal health record, health-related quality of life

## Abstract

**Background:**

There is a strong will and need to find alternative models of health care delivery driven by the ever-increasing burden of chronic diseases.

**Objective:**

The purpose of this 1-year trial was to study whether a structured mobile phone-based health coaching program, which was supported by a remote monitoring system, could be used to improve the health-related quality of life (HRQL) and/or the clinical measures of type 2 diabetes and heart disease patients.

**Methods:**

A randomized controlled trial was conducted among type 2 diabetes patients and heart disease patients of the South Karelia Social and Health Care District. Patients were recruited by sending invitations to randomly selected patients using the electronic health records system. Health coaches called patients every 4 to 6 weeks and patients were encouraged to self-monitor their weight, blood pressure, blood glucose (diabetics), and steps (heart disease patients) once per week. The primary outcome was HRQL measured by the Short Form (36) Health Survey (SF-36) and glycosylated hemoglobin (HbA1c) among diabetic patients. The clinical measures assessed were blood pressure, weight, waist circumference, and lipid levels.

**Results:**

A total of 267 heart patients and 250 diabetes patients started in the trial, of which 246 and 225 patients concluded the end-point assessments, respectively. Withdrawal from the study was associated with the patients’ unfamiliarity with mobile phones—of the 41 dropouts, 85% (11/13) of the heart disease patients and 88% (14/16) of the diabetes patients were familiar with mobile phones, whereas the corresponding percentages were 97.1% (231/238) and 98.6% (208/211), respectively, among the rest of the patients (*P*=.02 and *P*=.004). Withdrawal was also associated with heart disease patients’ comorbidities—40% (8/20) of the dropouts had at least one comorbidity, whereas the corresponding percentage was 18.9% (47/249) among the rest of the patients (*P*=.02). The intervention showed no statistically significant benefits over the current practice with regard to health-related quality of life—heart disease patients: beta=0.730 (*P*=.36) for the physical component score and beta=-0.608 (*P*=.62) for the mental component score; diabetes patients: beta=0.875 (*P*=.85) for the physical component score and beta=-0.770 (*P*=.52) for the mental component score. There was a significant difference in waist circumference in the type 2 diabetes group (beta=-1.711, *P*=.01). There were no differences in any other outcome variables.

**Conclusions:**

A health coaching program supported with telemonitoring did not improve heart disease patients' or diabetes patients' quality of life or their clinical condition. There were indications that the intervention had a differential effect on heart patients and diabetes patients. Diabetes patients may be more prone to benefit from this kind of intervention. This should not be neglected when developing new ways for self-management of chronic diseases.

**Trial Registration:**

ClinicalTrials.gov NCT01310491; http://clinicaltrials.gov/ct2/show/NCT01310491 (Archived by WebCite at http://www.webcitation.org/6Z8l5FwAM).

## Introduction

There is a strong will and need to find alternative models of health care delivery [1], driven by the ever-increasing burden of chronic diseases. To ensure adequate resources for the delivery of health care and to further improve the level of care, care-delivery models need to be changed in a way that patients themselves become more involved in their own care.

Home telemonitoring of chronic diseases seems to be a promising disease management approach with the potential to boost patients’ compliance with self-care, while bringing health care services closer to patients and, thus, resulting in improved quality of life. However, the evidence of the effectiveness of telemonitoring is contradictive and is dependent on the nature of the disease [2]. In a systematic review by Pare et al [2], it was found that telemonitoring improved glycemic control of diabetics, decreased blood pressure levels of hypertensive patients, and improved peak expiratory flows of patients with asthma and symptoms associated with the illness. However, the beneficial effect of telemonitoring was not associated with heart failure and the evidence is still contradictive. Meta-analyses conducted among heart failure patients from 2009 and 2011 conclude that there are beneficial effects of telemonitoring with linkage to improved survival and decreased hospitalizations [3,4]. However, since these meta-analyses, there have been two large-scale randomized controlled trials [5,6] failing to show the effectiveness of telemonitoring as concluded by Pare et al [2]. Correspondingly, the evidence of telemonitoring on improved glycemic control is contradictive. Typically, the observed reduction in hemoglobin A1c (HbA1c) has been 0.5% [7,8], raising a question of its clinical significance. Moreover, there have been studies that show nonsignificant changes in glycemic control among diabetics [9].

In chronic diseases the condition of a patient is highly dependent on their engagement of self-care and their ability to adhere to the management recommendations long term. For successful disease management, the education of a patient is important. However, the education-based interventions are by themselves insufficient [10]. Health coaching helps the patient to clarify his motivation to initiate and maintain change, offering a variety of perspectives and recognizing that numerous factors contribute to achieving goals [11]. Promising results have been obtained among type 2 diabetes patients in health coaching conducted by telephone [11]. However, the 1-year long health coaching by telephony to support self-care in chronic diseases (TERVA) trial, in which a health coaching approach was applied, failed to achieve most of the expected improvements in clinical measures [12]. Similar findings were found by Ruggiero et al [13]. In addition to the importance of self-management, patients and health care professionals need to share complementary knowledge in health care processes, which brings challenges and responsibility from both sides [14]. Telemonitoring provides a possibility for improved interaction. The combination of telemonitoring and remote monitoring has shown promising results among hypertensive patients [15].

The purpose of this study was to assess the benefits of a structured mobile phone-based health coaching program, supported by a remote monitoring system among chronically ill patients. We expected the intervention to improve patients’ engagement in self-management and to enrich the interaction between patients and health care professionals that would eventually result in improved quality of life and/or the clinical condition. Primarily, we hypothesized that we would see improved quality of life among patients suffering from heart disease or diabetes.

## Methods

### Study Design

The study was conducted as a two-armed randomized controlled trial (RCT) between February 2011 and December 2012 in the South Karelia Social and Health Care District (Eksote) in Finland. The trial was registered at ClinicalTrials.gov (NCT01310491). Eksote is responsible for arranging all primary and secondary health care for the inhabitants of eight municipalities, approximately 100,000 inhabitants. Patients with type 2 diabetes and patients suffering from heart disease were recruited to the study and assigned to either the control group or the intervention group. The study was approved by the Ethics Committee of the Social and Health Care District of South Karelia.

### Intervention

#### Overview

The intervention consisted of health coaching over mobile phones and self-monitoring of health parameters with the help of a remote patient monitoring (RPM) system.

#### Health Coaching

Each patient in the intervention group was assigned a personal health coach who called them at regular intervals—every 4 to 6 weeks. A comprehensive evaluation of the patient’s clinical, mental, and social condition was made during the first coaching call and small, achievable health behavior changes were agreed upon with the patient. A self-management plan was created based on the targeted changes. During the mobile phone calls that were planned to last for approximately 30 minutes, the health coach provided information, assistance, and support to the patients. The health coaching approach was provided by Pfizer Oy. The approach followed Wagner’s Chronic Care Model [16]—one of the key foundational constructs for the approach of chronic care management—and has been developed and tested earlier. The detailed structure of the health coaching program and the behavior change techniques involved are reported elsewhere [12].

#### Health Coach Recruitment

Health coaches and a health coach supervisor were recruited among the personnel of Eksote. Six coaches were recruited out of 13 applicants. Four of the recruits were working in outpatient care and two in a hospital. The selected coaches continued in their regular positions and worked as health coaches 1 day a week. The health coaches were trained to obtain the needed knowledge about Pfizer’s health coaching model, behavioral management skills, remote monitoring system, and trial procedures. The health coaching model was a solution-oriented working model where all patients received coaching based on their individual needs. For quality control and educational purposes, each health coach recorded some of the coaching calls, which were evaluated together with a behavioral science professional once in every 3 months. The equal quality of all health coaches was assured by continuous education and regular meetings, which all the health coaches and the trainer attended.

#### Remote Patient Monitoring

Each patient in the intervention group received a remote monitoring toolbox to be used in the trial. The toolbox consisted of a mobile phone with specific software, a mobile personal health record (PHR) app, and a set of measurement devices connected to the patient’s PHR account. The mobile PHR app was needed for manual and/or automatic reporting. All patients received a blood pressure meter, which was connectable to the mobile phone via Bluetooth. When the patients measured their blood pressure, the value was automatically transferred to the PHR using a binary short message service (SMS) text message. Other health parameters to be followed were body weight, blood glucose level for diabetics, and step count for heart disease patients. The patients were instructed to measure and send these values manually via the mobile phone to the PHR once a week. The health coaches and patients were able to see the patients’ measurements in the PHR and were advised to utilize them during health coaching phone calls. A self-management guide was given to the patients with the intention to increase their knowledge of their chronic disease.

#### Remote Patient Monitoring System

**Figure 1:**
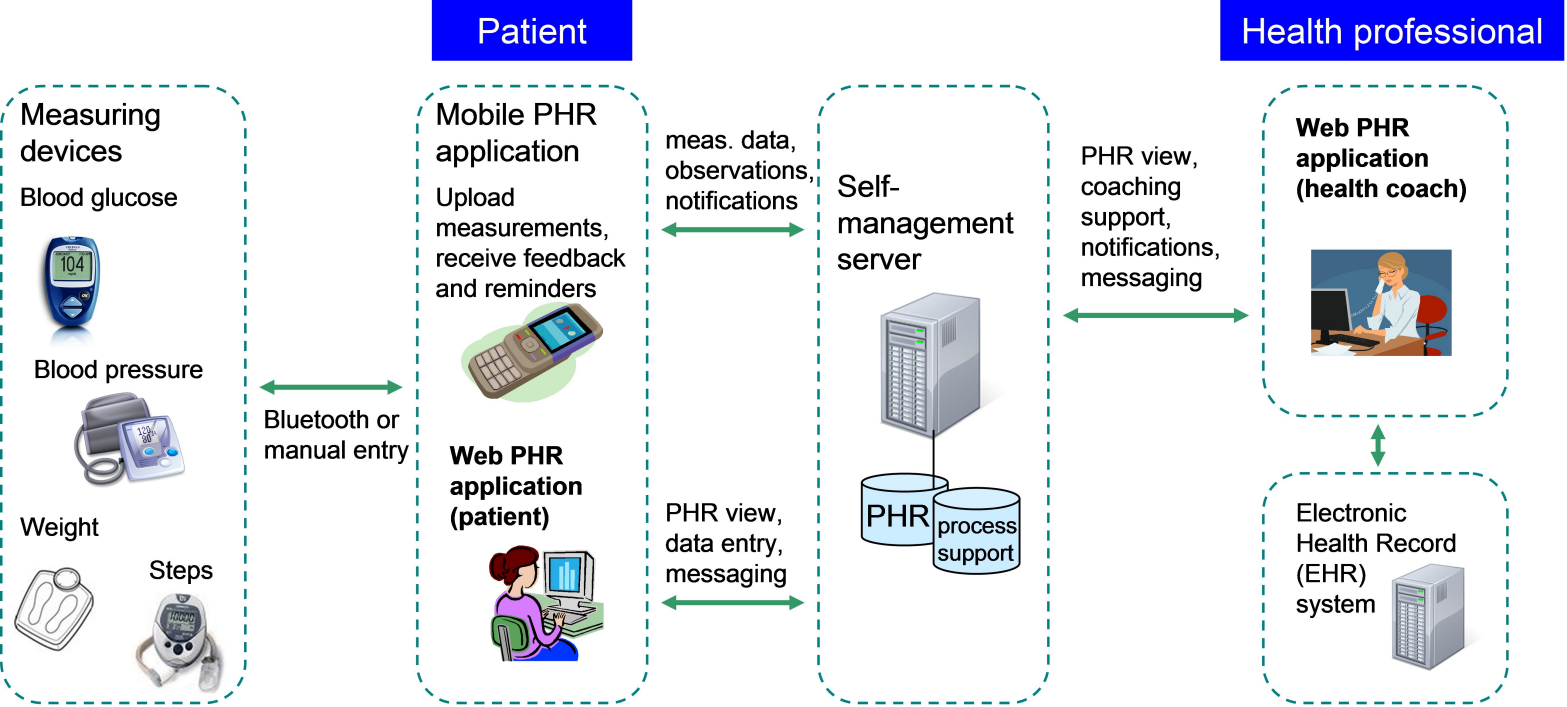
Technical architecture of the health coaching system supported with remote patient monitoring.

The intervention was supported by the RPM system, eClinic, provided by Medixine Ltd (Espoo, Finland) (see Figure 1). The self-management server is the central component of its architecture, providing services for the storing and accessing of information content (ie, RPM data) related to the self-management process. The RPM data included various types of information: health parameters registered by the corresponding measurement devices, personal care plan entered by the health coach in agreement with the patient, and data obtained from the electronic health record (EHR). The HTTPS protocol was used for sending all data from the mobile app to the server. The system underwent no major changes or updates during the trial.

### Standard Care

Patients assigned to the control group received the care they would have received in the absence of the study. As part of standard care, patients suffering from type 2 diabetes or heart disease receive a disease management information booklet at the time of diagnosis. Standard care includes laboratory tests taken once a year and 1 appointment or phone call by a nurse or doctor. Patients can contact health care services any time they feel they need to.

### Participants and Baseline Assessment

The patients’ eligibility was assessed primarily based on their diagnosis. The diabetic patients were recruited based on a diagnosis of type 2 diabetes mellitus and their glycosylated hemoglobin (HbA1c) level, which needed to be above 6.5% within 1 year prior to the screening. It was required that the patients had been diagnosed with diabetes at least 3 months earlier. The heart disease group consisted of patients with a diagnosis of ischemic heart disease, heart failure, or both. Other inclusion criteria for all patients were as follows: 18 years of age or older, ability to fill in questionnaires in Finnish, ability to use the RPM system and the devices provided, having adequate cognitive capacities to participate, and being able to walk.

Potential participants were screened using the electronic health record system of Eksote. EHRs cover information about citizens living in the health care district of South Karelia who have contacted health care services at least once. Invitation letters including information about the study were sent to eligible patients. Patients willing to participate signed an informed consent form before randomization. After that, the supervisor contacted each of the patients to schedule an appointment for a baseline visit. Randomization was done after the appointment was settled.

All patients who came in for the baseline visit were asked to fill in a demographic questionnaire and the Short Form (36) Health Survey (SF-36), version 2 [17], which measures health-related quality of life. At the baseline visit, a health coach measured the patient’s blood pressure, height (to the nearest 0.1 cm), weight (to the nearest 0.1 kg), and waist circumference (to the nearest 0.1 cm), and calculated their body mass index (BMI). Each patient’s medical history was reviewed based on the data in the EHR system. If laboratory tests were older than 2 months, new laboratory tests (ie, HbA1c, cholesterol, triglycerides) were done. At the end of the visit, the health coach checked that the required questionnaires were returned. If not, the patient was asked to fill out the questionnaire at home and send it to a nurse on the following day.

After 1 year following the baseline visit, all patients were invited to an end-point visit. The same procedures were conducted as they were during the baseline visit.

### Randomization

A stratified randomization design was used to assign patients to the control and intervention groups. Heart disease and diabetes patients were randomized into separate groups. Patients were further stratified into four subgroups according to their sex and dichotomized age—18 to 65 years versus older than 65 years. Within these subgroups, Excel-generated random numbers were produced. The allocation sequence was concealed from the research nurse by means of an opaque and sealed envelope until the baseline visit. During the baseline visit the envelope was opened and, according to its content, each patient was assigned to either group. The randomization was conducted by the Technical Research Centre of Finland (VTT).

### Outcome Variables

#### Short Form (36) Health Survey

The primary outcome for both disease groups was self-evaluated, health-related quality of life (HRQL) assessed based on the SF-36 health survey. Eight domains of HRQL and two summary component measures of physical and mental health were analyzed. Additionally, HbA1c level was another primary outcome for the diabetes patients.

#### Clinical Outcomes

Secondary outcomes were as follows: blood pressure (mmHg), weight (kg), waist circumference (cm), triglycerides (mmol/l), total cholesterol (mmol/l), low-density lipoprotein (LDL) (mmol/l), and high-density lipoprotein (HDL) (mmol/l). The selection of outcome variables was based on the use of a model for assessment of telemedicine applications [18]. However, this paper examines the first three out of the seven domains concentrating on the medical perspectives. Other domains, such as organizational and economic outcomes, will be reported in other articles in the future.

#### Adherence

Adherence to the health coaching was measured as the number and duration of health coaching calls. The duration of a call consisted of three parts—the time a nurse needed to prepare for a call (eg, familiarize herself with the self-measurement data of a patient), the duration of the actual coaching call, and the time a nurse needed to finalize the call (eg, notes, information delivery). Another perspective of the adherence measure was based on the frequency of home telemonitoring, measured as the total number of measurements made during the study and calculation of the number of weight, blood pressure, blood sugar, and step count reports. Both pre- and postprandial measurements were included in blood glucose reports.

### Statistical Analysis

We assumed we would see a difference of three points in the SF-36 scores between the intervention and control groups with a standard deviation of eight. The allocation ratio was unbalanced—approximately 2:1. The number of intervention patients was higher because we wanted to maximize the exposure to, and gain experience about, this new intervention. Defining a power of 80% and a Type I error rate of 5%, 163 intervention patients and 61 control patients were required. Predicting a dropout rate of up to 20%, at least 200 intervention patients and 75 control patients had to be randomized. The numbers were applied to both the heart disease group and diabetes group, resulting in 550 patients to be randomized in total. We used the *t* test as a basis for the power calculations, which is a conservative approach considering that repeated measures were available in the data, and thus more powerful tests could have been used.

The characteristics of dropout patients in terms of their baseline measures were explored using Student’s *t* tests and chi-square tests. All analyses were conducted separately for the diabetes and heart disease groups. The analysis of covariance (ANCOVA) was used to study whether the intervention and the control groups differed in terms of their outcome variables. The analyses were done by adjusting for the corresponding baseline level by adding the baseline measure as a covariate in the regression model. The 95% CIs and the corresponding *P* values were reported. Additionally, within-group changes from baseline to postintervention were analyzed using paired *t* tests.

Analyses were conducted following the intention-to-treat principle, meaning that all patients were analyzed in their original allocation group regardless of the extent to which they followed the intervention. No imputations were made to missing values, but missing values were excluded from the analyses. All reported *P* values were two sided. Analyses were conducted using IBM SPSS Statistics version 19.

## Results

### Patient Flow

**Figure 2:**
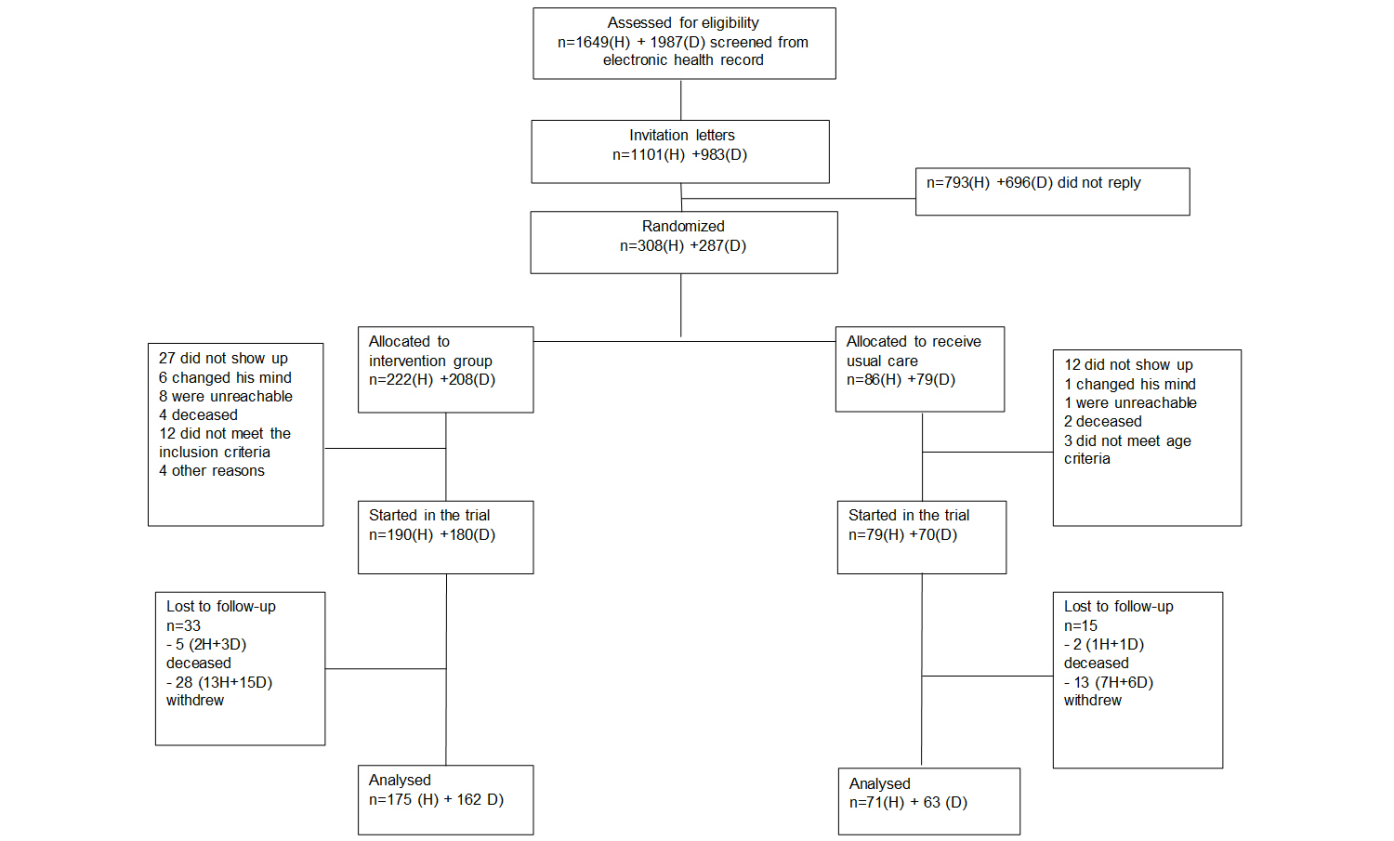
The patient flow within the trial. H: patients with a diagnosis of ischemic heart disease or heart failure, D: patients with a diagnosis of diabetes mellitus type 2 and HbA1c > 6.5%.

Figure 2 describes the progress of the trial. The electronic health records were utilized to screen patients with either heart disease or diabetes mellitus type 2. The diagnosis was either type 2 diabetes mellitus with HbA1c >6.5% or one of the following two heart diseases: ischemic heart disease or heart failure. The number of patients fulfilling the criteria was 1649 with heart disease diagnoses, and 1987 patients with diabetes diagnoses. Of these patients, 499 heart disease patients and 500 diabetes patients were randomly selected and received invitation letters in October 2010. The number of patients who refused to participate, changed their mind before the trial began, or did not show up at the baseline visit, was higher than expected. Therefore, the invitation procedure was repeated in November 2010 and August 2011 to achieve the predefined power for the pilot. In total, invitation letters were sent to 2084 patients, of which 28.02% (584) agreed to participate. Eventually, 595 patients were randomized and, of these, 519 patients (87.2%) attended the baseline visit. All participants filled out the baseline questionnaires before they were told into which group they were randomized.

There were 48 patients out of 519 (9.2%) lost to follow-up: 3 heart patients and 4 diabetes patients died, and 20 and 21 patients, respectively, withdrew from the trial without participating in the concluding visit. The baseline characteristics of the withdrawn patients were analyzed against patients who concluded the trial. Quitting was associated with the patients’ unfamiliarity with mobile phones—of the dropouts in the heart disease group, 85% (11/13) were familiar with mobile phones, whereas the corresponding percentage was 97.1% (231/238) among the rest of the patients (*P*=.02). Of the dropouts in the diabetes group, 88% (14/16) were familiar with mobile phones, whereas the corresponding percentage was 98.6% (208/211) among the patients who concluded the trial (*P*=.004). Among heart patients, withdrawal was also often associated with comorbidities—40% (8/20) of the dropouts had at least one comorbidity, whereas the corresponding percentage was 18.9% (47/249) among the rest of the patients (*P*=.04). There was no difference in the dropout rate between intervention and control groups. Eventually, 246 heart disease patients and 225 diabetes patients concluded the trial.

### Baseline Characteristics

Table 1 displays the baseline characteristic of patients separated according to their primary disease. Overall, patients were similar in the intervention group and in the control group in both disease groups. The mean age among heart patients was 69.1 (SD 9.1) years, and diabetes patients were slightly younger with a mean age of 66.2 (SD 8.6) years. The majority of patients were men in the heart disease group (178/269, 66.2%) and in the diabetes group (129/250, 51.6%). BMI was higher in the diabetes group than in the heart disease group, but BMI distribution was similar between the treatment arms. Over two-thirds of the patients (361/519, 69.6%) were retired. Approximately 8.1% (42/519) were smokers. The rate of missing values was clearly higher regarding smoking and alcohol questions compared to the other baseline questions. The high proportion of missing values regarding the alcohol question was explained by the fact that patients did not find a suitable option among the provided choices for answers. They told this to the nurse at the baseline visit, or it was written in the questionnaire that no proper choice was given because they did not use alcohol at all. The majority of the patients were familiar with mobile phones, and approximately half of the patients were familiar with computers. The most common comorbidities were diagnosed connective tissue disease, rheumatic disease, or chronic pulmonary disease. There were only a few patients with dementia or cerebrovascular disease.

### Short Form (36) Health Survey

Tables 2 and 3 show the baseline, postintervention, and change scores of HRQL—the eight dimensions of the HRQL assessment and the two summary scores. There were no significant differences between the control and intervention arms in either of the disease groups for any of the variables.

A total of 45 patients completed the baseline questionnaire at home and later sent it to the nurse. On average, these patients posted their questionnaires 5.3 (range 1 to 7) months after they started in the trial. To exclude the bias that the late responses may have caused, the analyses of HRQL were repeated without the late responses. The level of significance of the difference between the control and intervention groups remained above .1 in all variables. Thus, no change in the interpretation was observed.

The number of respondents varied from question to question. In the diabetes group, the number of respondents varied from 146 to 159 in the intervention group and 55 to 60 in the control group, depending on the questions, which is slightly less than was assumed in the pre hoc power calculations. The lower sample size leads to a post hoc power of .76 when using the *t* test framework. However, the magnitude of .80 was reached when using the ANCOVA framework. The predefined power was reached in the heart disease group.

**Table 1:** Baseline characteristics of the patients in the two disease groups.

Baseline characteristic		Heart disease patients (n=269), mean (SD) or n (%)		Diabetes patients (n=250), mean (SD) or n (%)	
		Control (n=79)	Intervention (n=190)	Control (n=70)	Intervention (n=180)
Sex (female), n (%)		25 (32)	66 (34.7)	30 (43)	81 (45.0)
Age (years), mean (SD)		68.1 (9.4)	69.6 (9.1)	65.5 (9.6)	66.6 (8.2)
BMI^a^(kg/m^2^), mean (SD)		28.1 (4.3)	28.6 (4.7)	30.9 (5.7)	31.1 (5.4)
**Education, n (%)**					
	Primary school or less	29 (37)	98 (51.6)	30 (43)	75 (41.7)
	Secondary or high school	31 (39)	59 (31.1)	24 (34)	65 (36.1)
	College/university or higher	9 (11)	24 (12.6)	12 (17)	27 (15.0)
	Missing	10 (13)	9 (4.7)	4 (6)	13 (7.2)
**Marital status, n (%)**					
	Never married	1 (1)	8 (4.2)	4 (6)	10 (5.6)
	Married/cohabitating	69 (87)	133 (70)	53 (76)	120 (66.7)
	Separated	3 (4)	24 (12.6)	4 (6)	25 (13.9)
	Widowed	5 (6)	23 (12.1)	9 (13)	22 (12.2)
	Missing	1 (1)	2 (1.1)	0 (0)	3 (1.7)
**Work status, n (%)**					
	Working	12 (15)	34 (17.9)	11 (16)	34 (18.9)
	Unemployed (able to work)	4 (5)	6 (3.2)	3 (4)	11 (6.1)
	Unemployed (unable to work)	0 (0)	5 (2.6)	0 (0)	5 (2.8)
	Retired	53 (67)	138 (72.6)	52 (74)	118 (65.6)
	Student	0 (0)	0 (0)	0 (0)	1 (0.6)
	Missing	10 (13)	7 (3.7)	4 (6)	11 (6.1)
**Smoking, n (%)**					
	Smoker	6 (8)	14 (7.4)	6 (9)	16 (8.6)
	Missing	17 (22)	27 (14.2)	14 (20)	23 (12.8)
**Alcohol, n (%)**					
	5-7 days a week	2 (3)	6 (3.2)	2 (3)	5 (2.8)
	1-4 days a week	21 (27)	40 (21.1)	13 (19)	34 (18.9)
	Monthly	14 (18)	47 (24.7)	11 (16)	37 (20.6)
	Less than monthly	18 (23)	52 (27.4)	23 (33)	65 (36.1)
	Missing	24 (30)	45 (23.7)	21 (30)	39 (21.7)
**Familiar with PC** ^b^ **use, n (%)**					
	Familiar	41 (52)	102 (53.7)	41 (59)	102 (56.7)
	Missing	10 (13)	14 (7.4)	8 (11)	14 (7.8)
**Familiar with mobile phone use, n (%)**					
	Familiar	69 (87)	173 (91.1)	61 (87)	161 (89.4)
	Missing	8 (10)	10 (5.3)	9 (13)	14 (7.8)
**Comorbidities, n (%)**					
	Heart diseases	79 (100)	190 (100)	15 (21)	47 (26.1)
	Cerebrovascular disease	0 (0)	4 (2.1)	3 (4)	9 (5.0)
	Chronic pulmonary disease, including COPD^c^	8 (10)	22 (11.6)	12 (17)	19 (10.6)
	Connective tissue disease or rheumatic disease	8 (10)	30 (15.8)	9 (13)	36 (20.0)
	Diabetes	18 (23)	46 (24.2)	70 (100)	180 (100)
	Cancer	7 (9)	17 (8.9)	4 (6)	12 (6.7)
	Other^d^	49 (62)	111 (58.4)	52 (74)	135 (75.0)
	No comorbidities	16 (20)	39 (20.5)	14 (9)	23 (6.2)

^a^BMI: body mass index

^b^PC: personal computer

^c^COPD: chronic obstructive pulmonary disease

^d^Hypertension is the most common “other” comorbidity.

**Table 2:** The baseline, postintervention, and change scores in the eight dimensions of the health-related quality-of-life assessments and in the two summary scores for heart disease patients.

Assessment	Control scores				Intervention scores				Between-group difference, beta (95% CI)	*P* ^a^
	n	Base- line	Post^b^	Change (95% CI)	n	Base- line	Post	Change (95% CI)		
Physical component score	68	40.3	40.7	0.39 (-0.72, 1.49)	162	39.5	40.8	1.25 (0.29, 2.22)	0.730 (-3.00, 1.78)	.36
Mental component score	68	50.5	51.0	0.55 (-1.53, 2.58)	162	50.4	50.3	-0.05 (-1.47, 1.37)	-0.608 (-6.19, 6.26)	.62
Physical functioning (PF)	68	64.9	66.1	1.16 (-1.77, 4.09)	170	62.7	64.1	1.42 (-0.82, 3.67)	0.02 (-3.89, 3.93)	.99
Role-physical (RP)	68	60.7	63.5	2.79 (-1.84, 7.42)	168	58.9	62.1	3.16 (-0.58, 6.90)	-1.72 (-6.09, 5.75)	.95
Bodily pain (BP)	68	57.2	57.9	0.70 (-3.27, 4.66)	171	56.4	59.9	3.51 (0.58, 6.44)	2.59 (-2.34, 7.51)	.30
General health (GH)	68	48.7	49.2	0.56 (-2.93, 4.05)	171	47.7	50.3	2.60 (0.36, 4.84)	1.77 (-2.06, 5.61)	.36
Vitality (VT)	68	57.1	56.9	-0.25 (-4.71, 4.22)	165	56.3	56.8	0.48 (-2.03, 3.00)	0.52 (-4.03, 5.06)	.82
Social functioning (SF)	68	80.1	80.0	-0.18 (-4.93, 4.56)	171	78.9	79.8	0.88 (-2.15, 3.90)	0.585 (-4.44, 5.61)	.82
Role-emotional (RE)	67	72.5	75.4	2.86 (-2.63, 8.35)	168	71.2	73.0	1.74 (-1.74, 5.22)	1.54 (-7.42, 4.34)	.61
Mental health (MH)	68	77.3	77.9	0.64 (-2.92, 4.21)	164	77.4	77.2	-0.23 (-1.47, 1.37)	-0.80 (-5.00, 3.36)	.70

^a^ *P* values show the level of statistical significance between the treatment arms.

^b^Postintervention score.

**Table 3:** The baseline, postintervention, and change scores in the eight dimensions of the health-related quality-of-life assessments and in the two summary scores for diabetes patients.

Assessment	Control scores				Intervention scores				Between-group difference, beta (95% CI)	*P* ^a^
	n	Base- line	Post^b^	Change (95% CI)	n	Base- line	Post	Change (95% CI)		
Physical component score	55	41.5	42.0	0.51 (-1.19, 2.21)	146	42.6	43.2	0.53 (-0.40, 1.47)	0.875 (0.80 9, 0.95)	.85
Mental component score	56	50.1	52.0	1.84 (0.02, 3.71)	148	50.2	51.2	1.06 (-0.42, 2.53)	-0.77 (-3.15, 1.61)	.52
Physical functioning (PF)	58	64.9	66.0	1.09 (-2.87, 5.06)	157	68.1	68.2	0.17 (-1.83, 2.17)	-0.715 (-4.74, 3.13)	.73
Role-physical (RP)	58	65.2	68.4	3.23 (-2.81, 9.27)	156	65.7	68.8	3.11 (-0.45, 6.68)	-0.036 (-6.19, 6.26)	.99
Bodily pain (BP)	58	55.3	58.8	3.52 (-0.94, 7.98)	159	62.4	62.2	-0.18 (-3.05, 2.68)	-2.02 (-7.20, 3.13)	.44
General health (GH)	60	49.2	50.6	1.34 (-1.48, 4.17)	159	50.1	53.6	3.47 (1.04, 5.89)	2.34 (-1.72, 6.41)	.26
Vitality (VT)	58	52.9	58.1	5.21 (1.29, 9.19)	149	57.6	58.6	0.98 (-1.88, 3.83)	-2.98 (-7.78, 1.83)	.22
Social functioning (SF)	60	79.4	83.3	3.96 (-0.18, 8.10)	157	80.0	81.1	1.19 (-2.05, 4.44)	-2.54 (-7.70, 2.61)	.33
Role-emotional (RE)	59	74.3	78.1	3.81 (-1.72, 9.35)	157	74.7	78.7	3.93 (0.26, 7.60)	0.30 (-5.50, 6.10)	.92
Mental health (MH)	58	76.5	78.5	2.07 (-1.80, 5.93)	149	76.7	77.5	0.87 (-1.75, 3.50)	-1.12 (-5.43, 3.19)	.61

^a^ *P* values show the level of statistical significance between the treatment arms.

^b^Postintervention score.

### Clinical Outcomes

Tables 4 and 5 display the baseline, postintervention, and change scores in the anthropometric and laboratory measures, and the comparison between the treatment arms in both disease groups. In the heart disease group, there was no difference between the treatment arms in any of the variables. However, there was a significant within-group decrease in waist circumference (*P*=.02), systolic blood pressure (*P*<.001), and LDL-cholesterol (*P*<.001) in the intervention group. Also, in the control group, LDL-cholesterol decreased significantly (*P*<.001), as did systolic blood pressure (*P*<.001).

Among diabetics, there was a significant difference between the treatment arms in waist circumference (*P*=.01). In the intervention group, there was a significant decrease in weight (*P*=.02), waist circumference (*P*<.001), systolic blood pressure (*P*<.001), diastolic blood pressure (*P*=.007), and LDL-cholesterol (*P*<.001). In the control group, systolic blood pressure and LDL-cholesterol decreased significantly (*P*=.02 and *P*<.001, respectively).

### Adherence

Out of 190 heart disease and 180 diabetes patients, 186 (97.9%) and 177 (98.3%) patients, respectively, received at least one health coach call. The average number of calls per patient was 8.7 (SD 1.6) in the heart disease patient group and 8.5 (SD 1.9) in the diabetes group. The difference between the disease groups was not significant (*P*=.40). The mean duration of a coaching call was 20.1 (SD 8.0) minutes in the heart disease group and 19.2 (SD 8.1) minutes in the diabetes group, with a significant between-group difference (*P*=.004). The mean time consumed by the nurse for the preparation of calls was 3.5 (SD 2.5) minutes in the heart disease group and 4.2 (SD 3.2) minutes in the diabetes group, and the between-group difference was significant (*P*<.001). The time consumed by the nurse after the coaching calls among heart disease and diabetes patients was 3.8 (SD 3.0) and 4.5 (SD 3.6) minutes, respectively, with a significant between-group difference (*P*<.001).

The median number of all self-measurements reported through mobile phones was 209 (interquartile range [IQR] 124-324) among heart patients and 217 (IQR 104-346) among diabetes patients. The median number for heart disease group-specific monitoring parameters per patient were the following: 18 (IQR 2-40) weight reports, 18 (IQR 4-43) step counts, 57 (IQR 36-89) blood pressure reports, and 42 (IQR 12-67) blood glucose reports—6 patients made blood glucose monitoring reports. The median number for diabetes group-specific monitoring parameters per patient were the following: 15 (IQR 3-39) weight reports, 15 (IQR 5-31) step counts, 56 (IQR 28-80) blood pressure reports, and 47 (IQR 20-89) blood glucose reports, including pre- and postprandial sugar. In the heart disease group and in the diabetes group, 174 out of 190 (91.6%) and 171 out of 180 (95.0%) patients, respectively, adhered to the self-monitoring intervention to the extent that they sent at least one report of any kind during the follow-up. Among 190 heart disease patients, 136 (71.6%) sent at least one weight measurement, 173 (91.1%) sent at least one blood pressure measurement, 6 (3.2%) sent at least one blood glucose measurement, and 118 (62.1%) sent at least one step count report. Out of 180 diabetes patients, the corresponding numbers were 119 (66.1%) for weight, 170 (94.4%) for blood pressure, 126 (70.0%) for blood glucose, and 13 (7.2%) for step count.

**Table 4:** Baseline, postintervention, and change scores in clinical outcomes for the heart disease group.

Clinical outcome	Control scores				Intervention scores				Between-group difference, beta (95% CI)	*P* ^a^
	n	Baseline	Post^b^	Change (95% CI)	n	Baseline	Post	Change (95% CI)		
Weight	70	79.9	79.1	-0.84 (-1.85, 0.16)	170	81.4	81.5	0.04 (-0.67, 0.76)	0.934 (-0.34, 2.21)	.15
Waist	65	97.6	98.7	1.10 (-1.65, 3.85)	160	101.5	100.6	-0.88 (-1.61, -0.16)	-1.518 (-3.57, 0.53)	.15
Systolic	68	144.4	138.0	-6.36 (-10.7, -2.01)	161	145.5	140.1	-5.43 (-8.12, -2.75)	1.587 (-2.51, 5.68)	.45
Diastolic	67	81.1	80.9	-0.18 (-2.81, 2.45)	161	82.3	82.1	-0.27 (-1.95, 1.41)	0.468 (-2.24, 3.18)	.73
Total cholesterol	68	4.13	4.05	-0.08 (-0.25, 0.09)	168	4.06	4.01	-0.05 (-0.17, 0.06)	0.009 (-0.168, 0.185)	.92
HDL^c^	68	1.23	1.26	0.03 (-0.02, 0.08)	168	1.29	1.31	0.02 (-0.01, 0.06)	-0.018 (-0.086, 0.05)	.87
LDL^d^	68	2.56	2.21	-0.36 (-0.51, -0.21)	168	2.50	2.16	-0.34 (-0.43, -0.24)	-0.008 (-0.15, 0.13)	.91
Triglycerides	68	1.43	1.32	-0.12 (-0.27, 0.03)	168	1.37	1.35	-0.01 (-0.13, 0.08)	0.071 (-0.08, 0.22)	.36

^a^ *P* values show the level of statistical significance between the treatment arms.

^b^Postintervention score

^c^HDL: high-density lipoprotein

^d^LDL: low-density lipoprotein

**Table 5:** Baseline, postintervention, and change scores in clinical outcomes for the diabetes group.

Clinical outcome	Control scores				Intervention scores				Between-group difference, beta (95% CI)	*P* ^a^
	n	Baseline	Post^b^	Change (95% CI)	n	Baseline	Post	Change (95% CI)		
HbA1c^c^	61	7.20	7.36	0.18 (-0.02, 0.35)	156	7.25	7.29	0.04 (-0.09, 0.17)	-0.106 (-0.33, 0.11)	.34
Weight	60	88.9	88.6	-0.30 (-1.21, 0.60)	153	89.6	88.7	-0.90 (-1.71, -0.22)	-0.566 (-1.86, 0.73)	.39
Waist	57	107.4	107.1	-0.29 (-1.47, 0.90)	143	107.8	105.8	-2.03 (-2.76, -1.29)	-1.711 (-3.042, -0.38)	.01
Systolic	60	151.9	147.8	-4.12 (-7.43, -0.81)	148	155.4	149.3	-6.10 (-9.10, -3.09)	-0.196 (-4.57, 4.18)	.93
Diastolic	60	86.7	84.6	-2.08 (-4.50, 0.34)	148	89.2	86.6	-2.61 (-4.50, -0.72)	0.668 (-2.18, 3.52)	.65
Total cholesterol	60	4.36	4.19	-0.16 (-0.35, 0.03)	153	4.35	4.25	-0.1 (-0.23, 0.04)	0.065 (-0.15, 0.28)	.54
HDL^d^	60	1.26	1.29	0.03 (-0.05, 0.12)	156	1.24	1.26	0.02 (-0.01, 0.05)	0.005 (-0.054, 0.064)	.61
LDL^e^	60	2.66	2.27	-0.39 (-0.55, -0.23)	156	2.74	2.35	-0.40 (-0.51, -0.28)	0.037 (-0.19, 0.20)	.66
Triglycerides	59	1.78	1.89	0.11 (-0.14, 0.36)	154	1.70	1.71	0.01 (-0.10, 0.10)	-1.22 (-0.32, 0.09)	.25

^a^ *P* values show the level of statistical significance between the treatment arms.

^b^Postintervention score

^c^HbA1c: hemoglobin A1c

^d^HDL: high-density lipoprotein

^e^LDL: low-density lipoprotein

## Discussion

### Principal Findings

This study evaluated whether health coaching, supported with home telemonitoring, improved health-related quality of life and/or the clinical condition of type 2 diabetes patients and heart disease patients after 12 months. The intervention failed to improve patients’ quality of life or their clinical condition. Patients received regular health coaching calls throughout the study and the majority of the patients adhered to the home telemonitoring plan and frequently monitored at least one of the required health parameters.

The intervention showed a statistically significant difference only in waist circumference among type 2 diabetics. However, due to the lack of consistency in other variables, this finding is likely a result of multiple tests conducted in this study rather than true a difference between the study groups. Multiple testing increases the likelihood of false positive discoveries and this should be acknowledged when interpreting the findings. In addition, blood pressure and cholesterol levels showed beneficial trends for all patients. Overall, the improvements in clinical variables were more apparent in the type 2 diabetes group than in the heart disease patient group.

There were 48 out of 519 patients (9.2%) that were lost to follow-up. We found that unfamiliarity with mobile phones and poor health status measured as a result of the presence of comorbidities were associated with withdrawal. These findings highlight the importance of offering and targeting interventions to an audience with the appropriate skills. eHealth literacy is a prerequisite for the success of eHealth interventions and should be appropriately accounted for. Electronic health tools provide little value if the intended users lack the skills to effectively engage with them [19]. As suggested by Cruz et al [20], the patient skills and acceptance of the technology should be measured prior to its implementation. Appropriate skills are also required on the professional side. A recent study evaluating the use of email in the communication between the primary health care system and general practitioners showed that the easier the general practitioners thought the email system to be, the more they used it [21]. In our study, six nurses were specifically trained for health coaching and to actively utilize the RPM system as part of the care.

The positive changes in patients’ clinical conditions in both study groups emphasize the well-known fact that control patients improve their lifestyles as a consequence of being involved in a trial, even if they are not subjected to the actual intervention. Some of the control group patients were disappointed for not being randomized into the intervention group and they decided to take better care of themselves. Regarding disease-specific effects, we found that diabetes patients who received the intervention improved their health status among several health parameters. The findings were not verified by testing statistical interaction of group and disease variables, but the results in Table 5 showed significant within-group reductions in patients’ weight, waist circumference, blood pressure, and LDL. We can speculate whether diabetes patients are more prone to benefit from this kind of intervention. Similarly, Pare et al reported that telemonitoring was associated with a decline in hemoglobin and better blood glucose control, but clinical effects on the condition of patients suffering from cardiac problems were not as evident [2]. Signals reflecting the state of diabetes are not apparent. Even the symptoms of the worsening condition of a patient may stay unrecognized. Therefore, the importance of self-management as a part of diabetes care should be emphasized. The utilization of self-management in health care is a good direction to take, as it was shown by Rose et al [22] that there is a risk of general practitioners, who are sensitive to patients’ low self-efficacy in blood glucose monitoring, taking over the monitoring role, and inadvertently reducing self-management. Furthermore, a recent study showed that the significant improvements in HbA1c achieved during a 6-month trial of home telemonitoring, combined with active medication management, were sustained for at least that same 6 months [23].

Patients adhered to home telemonitoring in terms of measuring their blood pressure. Assuming the duration of the trial was approximately 12 months, 52 parameters were expected to be reported. Heart disease and diabetes patients respectively produced 55 and 57 blood pressure measurements on average. Across other health parameters, the monitoring frequency varied from 15 to 42. Patient groups seemed not to differ from each other in terms of monitoring frequency. Some patients had a lack of skills in using remote monitoring devices or they had technical problems, which reduced the number of remote monitoring measurements. Health coaching was realized as planned. The expected number of health coaching calls was between 9 and 12, with 4 to 6 weeks calling frequency. The number of health coaching calls was 8.7 and 8.6 in the heart disease and diabetes group, respectively. Our health coaching model was solution oriented. All coaching calls were tailored to the individual needs that affected variation to the call durations. Few patients had lengthy hospital stays, which affected the number of health coaching calls. The number and duration of health coaching calls were significantly different between the disease groups. The low level of significance was likely due to a small standard deviation in the call duration. A 1-minute difference, as seen in the call duration, has no practical relevance.

The low inclusion criteria in terms HbA1c for diabetic patients posed a limitation on this study. For inclusion, a diabetic patient was required to have an HbA1c higher than 6.5%. On average, the HbA1c levels were 7.2%, showing that there was little room for improvement.

A lack of social support was a potential factor that may have influenced the negative findings of this study. Receiving real-time social support may help people to stay engaged and feel supported, which is important in order to initiate and maintain improvements in health-related behaviors [24]. Another appealing approach to keep patients motivated, specifically those involved with self-monitoring of their health parameters, is the utilization of active assistance technology. Active assistance technology involves automatic processing of health or behavior data and delivers automatic tailored messages to users [25]. Results in this field have been promising, including work by Quinn et al [26], Charpentier et al [27], and Orsama et al [28]. As Bock et al [29] have recently shown, in order to produce successful mHealth apps with lasting effects, it is important to obtain user input throughout development. In our study, the patients were contacted every 4 to 6 weeks. An automatic feedback system, based on their self-monitored health parameters, could have kept patients motivated and informed by the delivery of individualized feedback with a coaching perspective.

### Conclusions

In conclusion, this study failed to show a beneficial effect of health coaching supported by telemonitoring on patients’ quality of life or their clinical status. However, we do not yet know the long-lasting benefits of the intervention. There were indications that the intervention had a differential effect on heart disease patients and diabetes patients. Diabetes patients may be more prone to benefit from this kind of intervention. This should not be neglected when developing new ways for self-management of chronic diseases.

## Multimedia Appendix 1

CONSORT-EHEALTH checklist V1.6.2 [30].

## Abbreviations

ANCOVA: analysis of covariance
BMI: body mass index
BP: bodily pain
CIP: Competitiveness and Innovation framework Programme
COPD: chronic obstructive pulmonary disease
D: patients with a diagnosis of diabetes mellitus type 2 and HbA1c > 6.5% (in Figure 2)
EHR: electronic health record
Eksote: South Karelia Social and Health Care District
GH: general health
H: patients with a diagnosis of ischemic heart disease or heart failure (in Figure 2)
HbA1c: hemoglobin A1c, glycosylated hemoglobin
HDL: high-density lipoprotein
HRQL: health-related quality of life
ICT PSP: Information and Communication Technologies Policy Support Program
IQR: interquartile range
LDL: low-density lipoprotein
MH: mental health
PC: personal computer
PF: physical functioning
PHR: personal health record
RCT: randomized controlled trial
RE: role-emotional
RP: role-physical
RPM: remote patient monitoring
SF: social functioning
SF-36: Short Form (36) Health Survey
SMS: short message service
TERVA: health coaching by telephony to support self-care in chronic diseases
VT: vitality
VTT: Technical Research Centre of Finland
