# Supplementary material for: Telemonitoring and Mobile Phone-Based Health Coaching Among Finnish Diabetic and Heart Disease Patients: Randomized Controlled Trial
Source: J Med Internet Res. 2015 Jun 17;17(6):e153. doi: 10.2196/jmir.4059 (PMC4526947; doi:10.2196/jmir.4059)
Supplement: Supplementary file 1 [file jmir_v17i6e153_app1.pdf]

CONSORT-EHEALTH Checklist V1.6.2 Report

(based on CONSORT-EHEALTH V1.6), available at [http://tinyurl.com/consort-ehealth-v1-6].

4059

Date completed

11/21/2014 8:24:44

by

Anna-Leena Vuorinen

The effect of telemonitoring and telephone-based health coaching on health-related quality of life and clinical measures among Finnish diabetic and heart disease patients: a randomized controlled trial

TITLE

1a-i) Identify the mode of delivery in the title

"telemonitoring"

1a-ii) Non-web-based components or important co-interventions in title

"and telephone-based health coaching "

1a-iii) Primary condition or target group in the title

among Finnish diabetic and heart disease patients

ABSTRACT

1b-i) Key features/functionalities/components of the intervention and comparator in the METHODS section of the ABSTRACT

"a structured telephone-based health coaching programme which was supported by a remote monitoring system!

1b-ii) Level of human involvement in the METHODS section of the ABSTRACT

"structured telephone-based health coaching"

1b-iii) Open vs. closed, web-based (self-assessment) vs. face-to-face assessments in the METHODS section of the ABSTRACT

"Patients were recruited by sending invitation to randomly selected patients using electronic health records."

"The primary outcome was the self-assessed health-related quality of life measured by SF 36 and the change of HbA1c among diabetic patients. The clinical measures assessed were blood pressure, weight, waist circumference, and lipid levels."

1b-iv) RESULTS section in abstract must contain use data

"267 heart patients and 250 diabetes patients started in the trial, of which 246 and 225 patients concluded the end point assessments, respectively".

" The withdrawal was associated with the patients' unfamiliarity with mobile phones: of the 41 dropouts, 84.6% (heart disease patients) and 87.5% (diabetes patients) were familiar with mobile phones whereas the corresponding percentages were 97.1% and 98.6%, respectively, among the rest of the patients (P=0.019 and P=0.004). Withdrawal was also associated with heart disease patients' comorbidities: 40% of the dropouts had at least one comorbidity whereas the corresponding percentage was 19% among the rest of the patients (P=0.038). "

"The intervention showed no statistically significant benefits over the current practice with regard to the quality of life (heart disease patients: β=0.730, P=0.358 for physical component score and β=-0.608, P=0.616 for mental component score, diabetes patients: β=0.875, P=0.853 for physical component score, β=-0.770, P=0.523 for mental component score) or Hba1c (diabetes patients: β=-0.106, P=0.336)."

1b-v) CONCLUSIONS/DISCUSSION in abstract for negative trials

Health coaching programme supported with telemonitoring did not improve heart disease patients' or diabetes patients' quality of life or their clinical condition.

INTRODUCTION

2a-i) Problem and the type of system/solution

"There is a strong will and need to find alternative models of health care delivery [1] driven by the ever-increasing burden of chronic diseases. To ensure adequate resources for the delivery of health care and to further improve the level of care, care-delivery models need to be changed in a way that patients themselves become more involved of their own care. "

"Home telemonitoring of chronic diseases seems to be a promising disease management approach with potential to boost patients' compliance with self-care while bringing health care services closer to patients. "

"For successful disease management, the education of a patient is important. However, the education-based interventions are by themselves insufficient [10]. Health coaching helps the patient to clarify his motivation to initiate and maintain change, offering a variety of perspectives and recognizing that numerous factors contribute to achieving goals [11]. "

2a-ii) Scientific background, rationale: What is known about the (type of) system

"The evidence on the effectiveness of telemonitong is contradictive and is dependent on the nature of the disease [2]. In a systematic review by Pare et al.[2] it was found that telemonitoring improved glycaemic control of diabetics, decreased blood pressure levels of hypertensive patients and improved peak expiratory flows of patients with asthma and symptoms associated with the illness. However, the beneficial effect of telemonitoring was not associated with heart failure and the evidence is still contradictive."

"Promising results have been obtained among type 2 diabetes patients in health coaching conducted by telephone [11]. However, the one-year lasting TERVA trial, in which health coaching approach was applied, failed to achieve the most of the expected improvements in clinical measures [12]. "

METHODS

3a) CONSORT: Description of trial design (such as parallel, factorial) including allocation ratio

The purpose of this study was to examine whether a structured telephone-based health coaching program, which is supported by a remote monitoring system, could be used to improve the quality of life and/or the clinical measures of type 2 diabetes and heart disease patients. We hypothesized to see an improvement in patients' quality of life.

3b) CONSORT: Important changes to methods after trial commencement (such as eligibility criteria), with reasons

No changes.

3b-i) Bug fixes, Downtimes, Content Changes

"The system underwent no major changes or updates during the trial."

4a) CONSORT: Eligibility criteria for participants

"The diabetic patients were recruited based on the diagnosis of Diabetes Mellitus type 2 and their glycated haemoglobin (HbAc1) level that was measured to be over 6.5% within one year prior to the screening. It was required that the diabetes had been diagnosed at least 3 months earlier. The heart disease group included patients with diagnosed ischemic heart disease or heart failure or both. Other inclusion criteria were: 18 years of age or older, ability to fill in questionnaires in Finnish, ability to use the RPM system and the devices provided, having adequate cognitive capacities to participate, and being able to walk."

4a-i) Computer / Internet literacy

Inclusion criteria: ability to fill in questionnaires in Finnish, ability to use the RPM system and the devices provided.

4a-ii) Open vs. closed, web-based vs. face-to-face assessments:

"Potential participants were screened using electronic health record (EHR) system of Eksote. EHR covers information about citizens living in the healthcare district of South Karelia who have contacted health care at least once. For eligible patients, invitation letters including information of the study were sent. Patients willing to participate signed an informed consent before randomization. After that, the supervisor contacted each of the patients to schedule an appointment for a baseline visit. Randomization was done after the appointment was settled."

4a-iii) Information giving during recruitment

For eligible patients, invitation letters including information of the study were sent.

4b) CONSORT: Settings and locations where the data were collected

"All patients who came in for the baseline visit were asked to fill in a demographic questionnaire and SF-36 version 2 Health Survey [15] that measures health-related quality of life."

4b-i) Report if outcomes were (self-)assessed through online questionnaires

"All patients who came in for the baseline visit were asked to fill in a demographic questionnaire and SF-36 version 2 Health Survey [15] that measures health-related quality of life."

4b-ii) Report how institutional affiliations are displayed

Not displayed.

5) CONSORT: Describe the interventions for each group with sufficient details to allow replication, including how and when they were actually administered

5-i) Mention names, credential, affiliations of the developers, sponsors, and owners

"The intervention was supported by the RPM system (eClinic) provided by Medixine Ltd. (Espoo, Finland) (Figure 1)."
"The health coaching approach was provided by Pfizer Oy."

5-ii) Describe the history/development process

"The health coaches were trained to obtain the needed knowledge about Pfizer's health coaching model, behavioural management skills, remote monitoring system, and trial procedures. For quality control and educational purposes, each health coach recorded some of the coaching calls, which were evaluated together with a behavioural science professional once in every three months."

5-iii) Revisions and updating

Information not available

5-iv) Quality assurance methods

"For quality control and educational purposes, each health coach recorded some of the coaching calls, which were evaluated together with a behavioural science professional once in every three months."

5-v) Ensure replicability by publishing the source code, and/or providing screenshots/screen-capture video, and/or providing flowcharts of the algorithms used

Not available

5-vi) Digital preservation

Not available

5-vii) Access

"The patients were instructed to measure and send these values manually via the mobile phone to the PHR once a week. The health coaches and patients were able to see the patients' measurements in the PHR and were advised to utilize them during health coaching phone calls."

5-viii) Mode of delivery, features/functionalities/components of the intervention and comparator, and the theoretical framework

"The health coaching approach followed Wagner's Chronic Care Model [14] – one of the key foundational constructs for the approach of chronic care management – and has been developed and tested earlier [12]."
"Each patient in the intervention group was assigned a personal health coach who called them at regular intervals (every four to six weeks). During the telephone calls, which were planned to last for approximately 30 minutes, the health coach provided information, assistance, and support to the patients. Detailed structure of the health coaching programme and the behaviour change techniques involved are reported elsewhere [12]. "
"All patients received a blood pressure meter, which was connectable to the mobile phone via Bluetooth. When the patients measured their blood pressure, the value was automatically transferred to the PHR using a binary text message (SMS). Other health parameters to be followed were body weight and blood glucose level for diabetics and step count for heart disease patients. The patients were instructed to measure and send these values manually via the mobile phone to the PHR once a week."

5-ix) Describe use parameters

"The patients were instructed to measure and send these values manually via the mobile phone to the PHR once a week."

5-x) Clarify the level of human involvement

Health coaches

5-xi) Report any prompts/reminders used

No prompts/reminders

5-xii) Describe any co-interventions (incl. training/support)

"As part of standard care, patients suffering from diabetes type 2 or heart disease receive a disease management information booklet when the disease is diagnosed. Furthermore, standard care includes laboratory tests taken once a year and one appointment or phone call by nurse a doctor."

6a) CONSORT: Completely defined pre-specified primary and secondary outcome measures, including how and when they were assessed

"The primary outcome for both disease groups was a change in the self-evaluated health-related quality of life assessed based on the SF-36 Health Survey. Eight domains of health-related quality of life and two summary component measures of physical and mental health were analysed. Additionally, Hba1c level was another primary outcome for the diabetes patients."
"Secondary outcomes were: blood pressure (mmHg), weight (kg), waist circumference (cm), triglycerides (mmol/l), total cholesterol (mmol/l), low-density lipoprotein (LDL) (mmol/l) and high-density lipoprotein (HDL) (mmol/l). The selection of outcome variables was based on the use of a model for assessment of telemedicine applications c MAST [16]."

6a-i) Online questionnaires: describe if they were validated for online use and apply CHERRIES items to describe how the questionnaires were designed/deployed

No online questionnaires

6a-ii) Describe whether and how "use" (including intensity of use/dosage) was defined/measured/monitored

Usage not analysed.

6a-iii) Describe whether, how, and when qualitative feedback from participants was obtained

Patients had a possibility to give feedback during the health coaching phone calls.

6b) CONSORT: Any changes to trial outcomes after the trial commenced, with reasons

No changes

7a) CONSORT: How sample size was determined

7a-i) Describe whether and how expected attrition was taken into account when calculating the sample size

"We assumed to see a difference of three points in the SF-36 scores between intervention and control group with standard deviation of eight. The allocation ratio was unbalanced (approximately 2:1). Defining power of 80% and 5% level of type I error, 163 intervention patients and 61 control patients were required.
"Predicting dropout rate of up to 20% at least 200 intervention patients and 75 control patients had to be randomized. "

7b) CONSORT: When applicable, explanation of any interim analyses and stopping guidelines

No interim analyses

8a) CONSORT: Method used to generate the random allocation sequence

"Within these subgroups, Excel-generated random numbers were produced."

8b) CONSORT: Type of randomisation; details of any restriction (such as blocking and block size)

"A stratified randomization design was used to assign patients to control and intervention groups. Heart disease and diabetes patients were randomized into separate groups. Patients were further stratified into four subgroups according to their sex and dichotomized age (18-65 years versus >65 years). "

9) CONSORT: Mechanism used to implement the random allocation sequence (such as sequentially numbered containers), describing any steps taken to conceal the sequence until interventions were assigned

"The allocation sequence was concealed from the research nurse by means of an opaque and sealed envelope until the baseline visit. During the baseline visit the envelope was opened and according to its content each patient was assigned to either in control group of intervention group."

10) CONSORT: Who generated the random allocation sequence, who enrolled participants, and who assigned participants to interventions

The randomization was conducted by VTT Technical Research Centre of Finland.

11a) CONSORT: Blinding - If done, who was blinded after assignment to interventions (for example, participants, care providers, those assessing outcomes) and how

11a-i) Specify who was blinded, and who wasn't

not blinded for practical reasons

11a-ii) Discuss e.g., whether participants knew which intervention was the "intervention of interest" and which one was the "comparator"

not blinded for practical reasons

11b) CONSORT: If relevant, description of the similarity of interventions

Not relevant

12a) CONSORT: Statistical methods used to compare groups for primary and secondary outcomes

"All analyses were conducted separately for the diabetes and heart disease groups. The analysis of covariance (ANCOVA) was used to study whether the intervention and the control group differed in terms of their outcome variables. The analyses were done by adjusting for the corresponding baseline level by adding the baseline measure as a covariate in the regression model. The 95% CI's and the corresponding p-values were reported. Additionally, within-group changes from baseline to post intervention were analysed using a paired t-test.
Analyses were conducted following the Intention-To-Treat (I-T-T) principle, meaning that all patients are analysed in their original allocation group regardless of the extent to which they followed the intervention."

12a-i) Imputation techniques to deal with attrition / missing values

"No imputations were made to missing values but missing values were excluded from the analyses. All reported p-values were two-sided. "

12b) CONSORT: Methods for additional analyses, such as subgroup analyses and adjusted analyses

"The characteristics of dropout in terms of their baseline measures were explored using Student's t-test and Chi-square test."

RESULTS

13a) CONSORT: For each group, the numbers of participants who were randomly assigned, received intended treatment, and were analysed for the primary outcome

"The number of patients fulfilling the criteria was 1649 with heart disease diagnosis and 1987 patients with diabetes diagnosis. Of these patients, 499 heart disease patients and 500 diabetes patients were randomly selected and received invitation letters in October 2010. The number of patients who refused to participate, changed their mind before the trial began, or did not show up at the baseline visit, was higher than expected. Therefore, the invitation procedure was repeated in November 2010 and August 2011 to achieve the predefined power for the pilot. In total, invitation letters were sent to 2084 patients of which 28% agreed to participate. Eventually, 595 patients were randomized and of these 519 patients (87.2 %) attended the baseline visit. All participants filled in the baseline questionnaires before they were told into which group they were randomized.

There were 48 patients lost to follow-up: three heart patients and four diabetes patients died and 20 and 21 patients, respectively, withdrew from the trial without participating in the concluding visit. "

13b) CONSORT: For each group, losses and exclusions after randomisation, together with reasons

"Eventually, 595 patients were randomized and of these 519 patients (87.2 %) attended the baseline visit. All participants filled in the baseline questionnaires before they were told into which group they were randomized. "

13b-i) Attrition diagram

attrition diagram not included

14a) CONSORT: Dates defining the periods of recruitment and follow-up

Patients received invitation letters in October 2010. The number of patients who refused to participate, changed their mind before the trial began, or did not show up at the baseline visit, was higher than expected. Therefore, the invitation procedure was repeated in November 2010 and August 2011 to achieve the predefined power for the pilot.

14a-i) Indicate if critical "secular events" fell into the study period

No critical events.

14b) CONSORT: Why the trial ended or was stopped (early)

Trial was not stopped

15) CONSORT: A table showing baseline demographic and clinical characteristics for each group

Table 1. Baseline characteristics separately for heart disease patients and diabetes patients.

15-i) Report demographics associated with digital divide issues

In addition to conventional characteristics patients were asked about their familiarity with mobile phone and computer.

16a) CONSORT: For each group, number of participants (denominator) included in each analysis and whether the analysis was by original assigned groups

16-i) Report multiple "denominators" and provide definitions

For each outcome the number of patients whose data was available was shown.

16-ii) Primary analysis should be intent-to-treat

only ITT conducted

17a) CONSORT: For each primary and secondary outcome, results for each group, and the estimated effect size and its precision (such as 95% confidence interval)

For the primary and secondary outcomes the group differences were evaluated in terms of beta, its 95% confidence interval and the p-value.

17a-i) Presentation of process outcomes such as metrics of use and intensity of use

No usage metrics. Only analysis of drop-outs included.

17b) CONSORT: For binary outcomes, presentation of both absolute and relative effect sizes is recommended

No binary outcomes.

18) CONSORT: Results of any other analyses performed, including subgroup analyses and adjusted analyses, distinguishing pre-specified from exploratory

Forty-five patients completed the baseline questionnaire at home and sent it later to the nurse. On average, these patients posted their questionnaires 5.3 (range:1 to 7) months after they started in the trial. To exclude the possible confounding effect, the analyses of SF-36 were repeated without the late responses. The level of significance of the difference between the control and intervention groups remained above 0.1 in all variables. Thus, no change in the interpretation was observed.

18-i) Subgroup analysis of comparing only users

Not analysed in this paper.

19) CONSORT: All important harms or unintended effects in each group

NO unexpected harms or effects.

19-i) Include privacy breaches, technical problems

19-ii) Include qualitative feedback from participants or observations from staff/researchers

"The high proportion of missing in alcohol question is explained by the fact that patients did not find a suitable option among the provided choices for answers. They told this to nurse at the baseline visit, or it was written in the questionnaire that no proper choice was given because they did not use alcohol at all. "

DISCUSSION

20) CONSORT: Trial limitations, addressing sources of potential bias, imprecision, multiplicity of analyses

20-i) Typical limitations in ehealth trials

"One limitation of this study is that we did not investigate the usage of monitoring devices or attendance on health coaching phone calls. The extent to which the patients adhered to the intervention certainly affects its efficacy. The research nurse reported that not all patients conducted monitoring but they wanted attend health coaching phone calls only. A subgroup analysis conducted among those who accepted and utilized the intervention actively could reveal information to be utilized in targeting future eHealth interventions. Additionally, in our trial the inclusion criteria for the HbA1c-level was rather low (> 6.5%). On average the Hba1c levels were 7.2% showing that there was no room for big improvement. "

21) CONSORT: Generalisability (external validity, applicability) of the trial findings

21-i) Generalizability to other populations

not discussed

21-ii) Discuss if there were elements in the RCT that would be different in a routine application setting

not discussed

22) CONSORT: Interpretation consistent with results, balancing benefits and harms, and considering other relevant evidence

22-i) Restate study questions and summarize the answers suggested by the data, starting with primary outcomes and process outcomes (use)

"This study evaluated whether health coaching supported with home telemonitoring improves quality of life and/or clinical condition of type 2 diabetes patients and heart disease patients during the 12 months follow-up "

22-ii) Highlight unanswered new questions, suggest future research

"Considering disease-specific effects we found that diabetes patients in the intervention group improved their status in several variables. This indicates that diabetes patients may be more prone to benefit from this kind of intervention. The findings were not verified by testing statistical interaction of group and disease variables but the result table showed significant within group changes, specifically apparent in the diabetics who received the intervention. "

Other information

23) CONSORT: Registration number and name of trial registry

clinicaltrials.gov Identifier: NCT01310491

24) CONSORT: Where the full trial protocol can be accessed, if available

Protocol not published

25) CONSORT: Sources of funding and other support (such as supply of drugs), role of funders

50% of the funding of this study was received from the European Comission CIP-ICT PSP-2009 as part of the Renewing Health Project. 50% of the funding was provided by Eksote.

X26-i) Comment on ethics committee approval

"The study was approved by the Ethics Committee of the Social and Health Care District of South Karelia "

x26-ii) Outline informed consent procedures

"Patients willing to participate signed an informed consent before randomization. After that, the supervisor contacted each of the patients to schedule an appointment for a baseline visit."

X26-iii) Safety and security procedures

X27-i) State the relation of the study team towards the system being evaluated

Tapio Jokinen is the chairman of the board of Medixine Oy which provided remote patient monitoring system.
